# Radiotherapy-induced toxicity in prostate cancer patients with hip prostheses

**DOI:** 10.1186/s13014-021-01975-3

**Published:** 2022-01-17

**Authors:** Andrea M. Fischer, Peter J. Hoskin

**Affiliations:** 1 Mount Vernon Cancer Centre, Rickmansworth Road, Northwood, HA6 2RN UK; 2 Division of Cancer Sciences, Faculty of Biology Medicine and Health, University of Manchester, Manchester, UK

**Keywords:** Radiotherapy, Prostate cancer, Hip prosthesis, Bladder and rectal toxicity

## Abstract

**Introduction:**

Acute and late toxicity was analysed for prostate cancer patients with bilateral hip prostheses, who received fixed field intensity modulated radiotherapy (IMRT). The aims were (1) to establish whether toxicity rates differed from those of a control group with normal hips, (2) to develop a volumetric modulated arc therapy (VMAT) approach for patients with prostheses and (3) to compare doses to bladder and rectum for the control group, prostheses group and VMAT replans for the prostheses group.

**Methods:**

Genitourinary (GU) and gastrointestinal (GI) toxicity was scored using Common Terminology Criteria for Adverse Events version 5.0. The incidence of grade 2 or worse (G2+) toxicity was compared using Fisher’s exact test. Dose volume histograms (DVHs) and mean doses to organs at risk (OARs) were compared using signed rank tests.

**Results:**

There were 17 patients in the prostheses group and 50 in the control group. Acute and late GU toxicity was similar. G2+ late GI toxicity incidence was 31% for the prostheses group and 14% for the control group (*p* = 0.14). Significant differences (*p* < 0.05) were seen between the OAR DVHs of the prostheses group who had IMRT and the control group for a range of intermediate doses. The rectum mean dose was significantly different (*p* < 0.001), but no difference was seen for the bladder mean dose (*p* = 0.08).

**Conclusions:**

No significant differences were seen in GU and GI toxicity incidence between patients with bilateral hip prostheses and a control group. The DVHs for bladder and rectum were significantly higher for patients with prostheses planned with IMRT. Replanning using a VMAT technique significantly reduced doses to the OARs, whilst maintaining good planning target volume coverage.

## Introduction

An aging population and improved cancer survival rates [1] mean that the number of patients with hip prostheses [2] at the time of radiotherapy or who subsequently have total hip arthroplasty [3] is increasing. Treating patients with hip prostheses is challenging [4] due to artefacts in computed tomography (CT) images making it hard to accurately delineate the clinical target volumes (CTVs) and organs at risk (OARs) [5], difficulties in determining the prosthesis structure and density needed for dose calculation [6, 7], and limitations associated with many dose calculation algorithms [8, 9]. With the exception of Monte Carlo and linear Boltzmann transport equations solvers [10], these algorithms are unable to correctly model beam attenuation by a high density prosthesis [11]. Furthermore they completely fail to capture lateral scatter peaks and the backscatter peak upstream of the prosthesis [12]. Underestimation of the dose by the treatment planning system (TPS) at the bone-prosthesis interface is of concern due to the known clinical effects of radiation on bone [13]. A recent study has seen a correlation between hip replacement dysfunction and mean dose to the arthroplastic femoral head in excess of 30 Gy [14].

In order to minimise uncertainties in the dose distribution, it is common to use a planning approach which minimises the proportion of the beam entering the planning target volume (PTV) having first passed through the prosthesis [15–18]. Due to an increased proportion of the beam entering the patient anteriorly and posteriorly, patients with hip prostheses may receive higher doses on average to their rectum and bladder, resulting in higher genitourinary (GU) and gastrointestinal (GI) toxicities, and this effect may be heightened in patients with bilateral prostheses.

The primary goal of this study was to determine whether the incidence of GU and GI toxicities following radiotherapy for prostate cancer differed between a group of patients with bilateral hip prostheses and a control group. Doses to the rectum and bladder were analysed and a comparison made between fixed field intensity modulated radiotherapy (IMRT) as used for treatment and volumetric modulated arc therapy (VMAT) plans for the group with prostheses.

## Materials and methods

### Patient characteristics

Data from 17 patients with bilateral hip prostheses treated for prostate cancer in a single UK centre between 2011 and 2018 was analysed. Fifty consecutive contemporary patients with no prostheses were identified for a control group, matching for age and treatment dates. All patients gave written consent for their data to be used for service evaluation. There was no evidence of pathological lymph nodes or bone metastases for any of the patients prior to treatment. None of the patients had received previous radiotherapy. The majority of patients received androgen deprivation therapy, see Table 1.

**Table 1:** Patient characteristics

	Group with bilateral hip prostheses	Control group
Sample size	17	50
Median age (years), range	77, 65–84	77, 64–83
T stage: ≤ T1c	2 (12%)	3 (6%)
T2a–T2c	7 (41%)	43 (86%)
≥ T3a	8 (47%)	4 (8%)
Androgen deprivation therapy		
None	2 (12%)	6 (12%)
< 6 months	1 (6%)	1 (2%)
6–12 months	4 (24%)	43 (86%)
> 12 months	10 (59%)	0 (0%)
Radiotherapy prescription		
57 Gy in 19 fractions	3 (18%)	8 (16%)
60 Gy in 20 fractions	14 (82%)	42 (84%)
Median CTV volume (cc), range	56.3, 21.8–144.1	47.1, 22.1–135.9
Bladder filling: empty	14 (82%)	39 (78%)
full	3 (18%)	11 (22%)
Median hip separation (cm), range	13.5 (11.2, 16.2)	12.8 (10.8,14.5)

### CT simulation and treatment planning

Patients were treated with an empty bladder, in the majority of cases (see Table 1), and had bowel preparation using enemas. Set up was verified using either cone beam computed tomography or fiducial markers viewed in an orthogonal pair of kilovoltage images. Plans were created on a CT scan acquired using either an Aquilion LB (Toshiba Medical Systems Corp, Ōtawara, Japan), Somatom Definition AS (Siemens Healthineers, Erlangen, Germany) or Somatom Sensation Open CT scanner (Siemens Healthineers, Erlangen, Germany). Fusion with a magnetic resonance image set was used to aid contouring for 10 out of 17 patients with prostheses. The CTV comprised the prostate and, where appropriate, the seminal vesicles. The nodes were not treated. The rectum, bladder and femoral heads (control group only) were contoured as OARs. For the patients with prostheses, streaking artefacts and missing data were outlined and the density overridden to 1 g/cm^3^. The prostheses were contoured and a hip planning avoidance volume (PAV) created using a 5 mm margin. The CTV to PTV margin was 5 mm posteriorly and 10 mm in all other directions.

All plans were created on the Eclipse TPS (Varian Medical Systems, Palo Alto, CA) using 6 MV photon beams. Final dose calculation was performed using the Anisotropic Analytical Algorithm (64 patients) or Pencil Beam Convolution (3 patients). Patients in the control group were planned using a VMAT approach with either a single full arc, or 2 partial arcs (260–100°), if there were concerns about the reproducibility of set up due to rectal size. Patients with bilateral hip prostheses were planned using fixed field IMRT with beams at 5–7 gantry angles. The gantry angles and the jaw positions were chosen, so that the beam did not enter the PTV through the hip PAVs. The collimator angles were adjusted to make best use of the beam.

Patients with bilateral hip prostheses were retrospectively replanned and the dose distributions compared to those of the clinical fixed field IMRT plans. Two VMAT arcs were used with two 30° avoidance sectors centred on the hip PAVs, over which no monitor units were delivered. In addition, dose objectives were used in the optimiser to prevent beam entry through the PAVs. These ensured that the multileaf collimators blocked beam entry through the PAVs outside the avoidance sectors. During plan evaluation each control point was examined from the beam’s eye view to ensure this had been achieved. The underestimation of dose close to the prostheses was not a concern for these patients, since the PTV was always > 1 cm from the prostheses.

All plans utilised the optimiser (Dose Volume Optimizer for IMRT plans and Progressive Resolution Optimizer for VMAT plans) to spare the OARs whilst maintaining PTV coverage. Local dose constraints are shown in Table 2. These were not met for all plans; plan approval was granted after a review of the dose volume histograms (DVHs) and 3D dose distribution, taking into account the relative geometry of the PTV and OARs. Patients were treated on Clinac 2100CD, Clinac iX and TrueBeam linear accelerators (Varian Medical Systems, Palo Alto, CA).

**Table 2:** Local dose constraints for PTV, bladder and rectum and the number of plans meeting these constraints

Local dose constraint	Number of plans meeting constraint		
	Control group	Prostheses group (IMRT clinical plan)	Prostheses group (VMAT replan)
PTV V95% > 98%	50 (100%)	16 (94%)	17 (100%)
PTV V105% < 2%	50 (100%)	17 (100%)	17 (100%)
Rectum V42Gy < 60%	49 (98%)	10 (59%)	16 (94%)
Rectum V50Gy < 50%	49 (98%)	14 (82%)	16 (94%)
Rectum V54Gy < 30%	49 (98%)	13 (76%)	16 (94%)
Rectum V58Gy < 15%	46 (92%)	16 (94%)	16 (94%)
Bladder V42Gy < 55%	43 (86%)	10 (59%)	14 (82%)
Bladder V50Gy < 40%	40 (80%)	9 (53%)	14 (82%)
Bladder V62Gy < 5%	41 (82%)	11 (65%)	14 (82%)

### Toxicity scoring

Patients were monitored weekly during treatment and 6 weeks post treatment to assess acute treatment related toxicities. Late toxicity was assessed between 2012 and 2020 once for each patient with median follow up times of 39 months (range 9–93 months) and 48 months (range 23–62 months) for the prostheses and control groups, respectively. All data was obtained by patient consultation, by either a radiographer, clinician or physicist. GU and GI toxicity scoring was carried out according to the National Cancer Institute Common Terminology Criteria for Adverse Events (CTCAE), version 5.0.

### Statistical analysis

Two-sided Fisher’s exact test was used to determine whether there was a significant difference in grade 2 or higher (G2+) toxicity between the two groups. Mean DVHs for the bladder and rectum were calculated with 95% confidence intervals for (1) the control group, (2) the clinical fixed field IMRT plans of the group with bilateral prostheses and (3) the retrospective VMAT plans of the prostheses group. The DVHs of the prostheses and control group were compared by the Mann–Whitney U test on the two sets of volume data (VxGy, the percentage of OAR volume receiving a dose of x Gy or greater) at each dose point. The DVHs for IMRT and VMAT plans for the prostheses group were compared using Wilcoxon matched pairs signed ranks test at each dose point with each pair equal to the volume statistics at that dose from the IMRT and VMAT plans for a specific patient. Differences in mean OAR doses between the groups were examined using the same tests. The dose response was also investigated by reallocating patients to 2 groups based on whether they had or had not experienced late G2+ toxicity and comparing the DVHs using the Mann–Whitney U test. All statistical testing was conducted in R (R Foundation for Statistical Computing, Vienna, Austria).

## Results

### Toxicities

The incidence of GU and GI toxicities is shown in Table 3 comparing rates of G2+ toxicity between the prostheses and control group. The most commonly observed acute G2 GU toxicities were a moderate increase in urinary frequency or nocturia (11 patients, 16%) and urinary retention requiring catheter placement (2 patients, 3%). Five patients (7%) started taking alpha blockers whilst on treatment and 10 patients (15%) reported taking alpha blockers at late follow up, having not taken them pre-treatment. At late follow up 4 patients (6%) had a new onset of incontinence with regular use of pads. Two patients (3%) experienced constipation requiring daily use of laxatives during and shortly after treatment. The most common late G2+ GI toxicities were rectal bleeding requiring medical intervention (5 patients, 7%) and constipation requiring daily use of laxatives (6 patients, 9%), where this hadn’t been indicated prior to treatment. One patient from the control group had G3 rectal bleeding needing several blood transfusions.

**Table 3:** Toxicity rates for the prostheses (top) and control group (bottom)

Prostheses group CTCAE grade	Acute GU *n* = 17 (%)	Acute GI *n* = 16^a^ (%)	Late GU *n* = 17 (%)	Late GI *n* = 16^a^ (%)
0	5 (29)	5 (31)	3 (18)	5 (31)
1	7 (41)	10 (63)	10 (59)	6 (38)
2	5 (29)	1 (6)	4 (24)	5 (31**)**

Control group CTCAE grade	*n* = 50 (%)	*n* = 50 (%)	*n* = 50 (%)	*n* = 50 (%)
0	2 (4)	19 (38)	28 (56)	31 (62)
1	36 (72)	29 (58)	12 (24)	12 (24)
2	12 (24)	2 (4)	10 (20)	6 (12)
3	0	0	0	1 (2)
*p* value	0.75	1.00	0.74	0.14

The *p* values from the Fisher’s exact test indicate whether there is a significant difference in grade 2 or higher toxicity between the groups

^a^One patient in the prostheses group had a stoma prior to irradiation

### Dosimetry

**Fig. 1:**
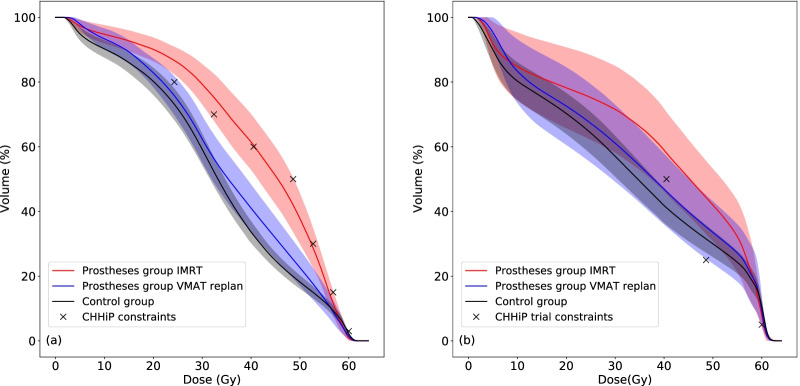
Mean rectal (**a**) and bladder (**b**) DVHs for the prostheses group, the control group and the prostheses group replanned using VMAT. The shaded regions correspond to the confidence intervals

**Fig. 2:**
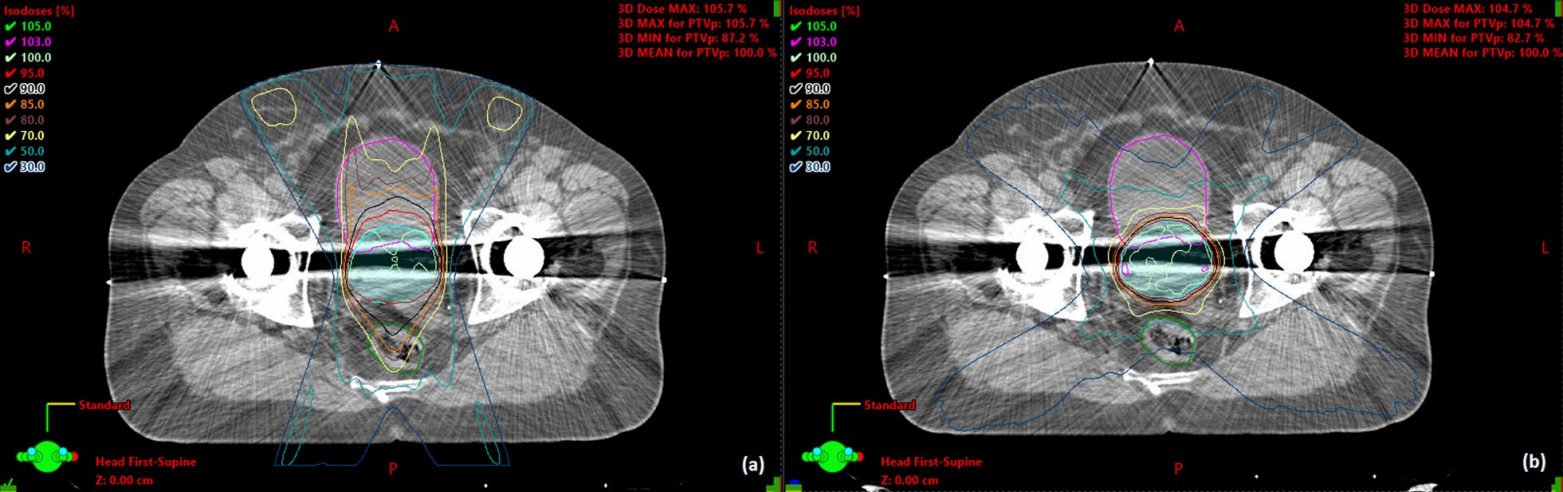
Dose distributions for **a** the clinically delivered IMRT plan and **b** the retrospective VMAT replan

A comparison between mean DVHs for the prostheses group, control group and the prostheses group replanned using VMAT are shown in Fig. 1. Statistically significant differences in mean doses to the OARs are indicated in Table 4. DVHs for the patients in the prostheses group and control group were significantly different (*p* < 0.05) for the rectum at V14Gy–V56Gy and for the bladder at V28Gy–V51Gy. Replanning the patients from the prostheses group using VMAT with avoidance sectors and dose objectives on the hip PAVs reduced the mean DVHs compared to the clinical IMRT plans for intermediate and high doses. These differences were statistically significant for the rectum at V8Gy–V57Gy and the bladder at V12Gy–V57Gy. At very low doses the mean DVH for the VMAT replan exceeded that of the IMRT plan; this was statistically significant at V2.5Gy–V5Gy for the rectum and V3Gy–V6Gy for the bladder. Since doses ≤ 10% of the prescription dose are of little interest clinically compared to intermediate and high doses, the OAR sparing provided by the VMAT plans is superior to that of the IMRT plans. This was not at the cost of reduced PTV coverage; all the replans achieved V95% ≥ 99% for the PTV. The number of plans meeting local rectum and bladder dose constraints is shown in Table 2 for each group. The pass rates are lower for the prostheses group compared to the control group, whereas the VMAT replans for the prostheses group are similar to the control group. Figure 2 shows an example of the dose distributions for IMRT and VMAT plans on the same patient.

**Table 4:** Mean rectum and bladder doses

Dose statistic	Mean value (95% confidence interval)			*p* value	
	Control	Hip prostheses, IMRT	Hip prostheses, VMAT replan	IMRT versus control	VMAT replan versus control
Rectum Mean dose (Gy)	33.5 (31.9, 35.0)	41.6 (39.1, 44.1)	35.6 (33.1, 38.1)	< 0.001	0.50
Bladder Mean dose (Gy)	33.9 (31.0, 36.8)	39.6 (33.5, 45.6)	35.9 (30.5, 41.2)	0.08	0.71

DVHs were also compared regrouping the patients into those who had late GI G2+ toxicity and those who didn’t. The mean DVH was higher for the group with G2+ toxicity and this was statistically significant for V50Gy–V59Gy. No statistically significant difference was seen comparing the DVHs for patients with and without late GU G2+ toxicity.

**Fig. 3:**
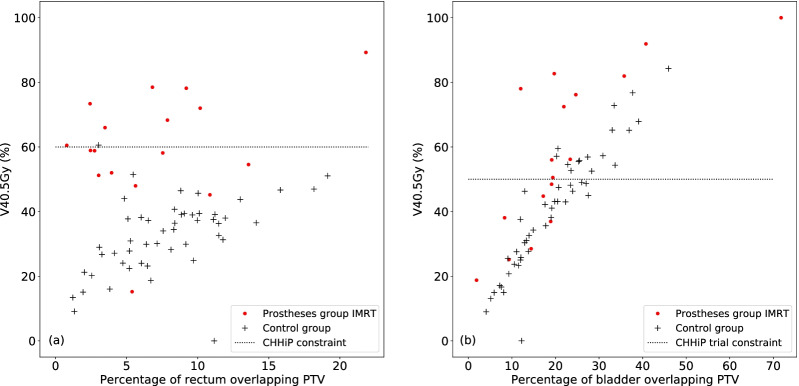
V40.5 Gy for **a** the rectum and **b** the bladder plotted against the OAR overlap with PTV as a percentage of the OAR volume for the prostheses group (clinically delivered IMRT plan) and the control group

Confounding factors age (see Table 1) and OAR positioning have been considered. The positioning of the OARs relative to the PTV was investigated by calculating the volume of the OAR that overlaps the PTV as a percentage of the total OAR volume. For the rectal volume, the percentage overlapping the PTV was 6.9 and 7.9% for the prostheses and control groups respectively and for the bladder volume, it was 22.2 and 19.8% for the prostheses and control groups respectively. Figure 3 shows the V40.5Gy dose statistic plotted against the percentage of OAR overlapping the PTV for all patients. There is an overall upwards trend in V40.5Gy as the overlap with the PTV increases. The V40.5Gy is in most cases higher for patients in the prostheses group when compared to patients in the control group with similar PTV overlaps. This effect is particularly marked for the rectum.

## Discussion

The incidence of G2+ GU toxicities is comparable between the group with bilateral prostheses and the control group. The incidence of G2+ late GI toxicity at 31% for the prostheses group is quite high, but only one G3+ event was observed which was in the control group. A previous study [17] reported a crude incidence of 9% for late GI G2+ RTOG adverse events in a mixed group of patients with unilateral and bilateral prostheses using VMAT with avoidance sectors.

The mean DVHs for rectum and bladder are significantly higher for a broad range of doses for the prostheses group planned with IMRT compared to the control group planned with VMAT. The mean rectal DVH for the prostheses group planned with IMRT exceeds 2 of the optimal and 1 of the mandatory constraints used in a large multicentre trial (CHHiP) [19], whereas the mean rectal DVH for the control group meets all 7 constraints. Replanning the prostheses patients using VMAT with 30° avoidance sectors and dose constraints on the hip PAVs in the optimiser significantly reduced doses to the OARs, giving similar quality plans to those of patients in the control group. Hence VMAT is recommended over fixed field IMRT for patients with bilateral hip prostheses.

Many studies have examined the relationship between OAR dose/volume parameters and toxicity. The QUANTEC group found that most dose/volume thresholds associated with a significant increase in late rectal toxicity involved lower volumes receiving higher doses [20]. The dose response data for the bladder was more heterogeneous [21]. A more recent review [22] examining tolerances for distinct GU and GI symptoms in prostate cancer patients, found consensus between studies on late rectal toxicity and a spread of rectal tolerances ranging from low to high doses, which were used to create a threshold DVH with 95% confidence intervals indicating regions with low and high risks of GI toxicity. The mean rectal DVH lies below this threshold and largely in the low risk region for the control group, largely above the threshold but not in the high risk region for the prostheses patients planned using IMRT and largely below the threshold but not in the low risk region for the prostheses patients retrospectively planned with VMAT. The data on thresholds for GU toxicities remains inconclusive.

This study is limited by the small sample size of patients with bilateral hip prostheses, since only large differences in the incidences of G2+ toxicity, if they existed, would be likely to be detected. For example, if the true G2+ toxicity incidence was 10% for patients without prostheses and 40% for patients with bilateral prostheses, the power would be 0.7. It was also not possible to consider different types of GU and GI symptoms separately or radiotherapy for other types of cancer. Outcomes were reported by either a clinician, radiographer or physicist and late toxicity assessed at a single time point only; studies suggest that the use of patient reported outcomes may have led to an increase in event rate [23, 24]. DVH data was taken from treatment plans and is expected to differ from the delivered DVH, mostly due to variations in OAR positioning and filling during a fractionated course of treatment [25, 26]. Factors other than dose that may affect a patient’s likelihood of developing late toxicity, such as pre-existing comorbidities, were not considered.

In conclusion no significant differences were observed in the incidence of acute and late GU and GI toxicity between the cohort with bilateral hip prostheses and control group. Rectum and bladder doses were significantly lowered for the patients with prostheses by using a VMAT approach with avoidance sectors and PAVs for the prostheses instead of fixed field IMRT.

## Data Availability

The datasets used and/or analysed during the current study are available from the corresponding author on reasonable request.

## Abbreviations

CT: Computed tomography
CTCAE: Common Terminology Criteria for Adverse Events
CTV: Clinical target volume
DVH: Dose volume histogram
GI: Gastrointestinal
GU: Genitourinary
IMRT: Intensity modulated radiotherapy
OAR: Organ at risk
PAV: Planning avoidance volume
PTV: Planning target volume
TPS: Treatment planning system
VMAT: Volumetric modulated arc therapy
